# Polyelectrolyte Microcapsule Stability: Non-Monotonic Layer-Dependent Desorption Kinetics of Poly(allylamine hydrochloride)

**DOI:** 10.3390/polym18060690

**Published:** 2026-03-12

**Authors:** Egor V. Musin, Alexey V. Dubrovskii, Aleksandr L. Kim, Sergey A. Tikhonenko

**Affiliations:** 1 Moscow Polytechnic University (Moscow Polytech), Bolshaya Semyonovskaya Str., 38, 107023 Moscow, Russia; eglork@gmail.com; 2 Institute of Theoretical and Experimental Biophysics, Russian Academy of Science, 3, Institutskaya Str., 142290 Pushchino, Moscow Region, Russia; dav198@mail.ru

**Keywords:** polyelectrolyte microcapsules, layer-by-layer assembly, poly(allylamine) desorption, core template effect, controlled release, ionic strength, shell stability

## Abstract

Polyelectrolyte microcapsules (PMCs) fabricated by layer-by-layer assembly require predictable shell stability for applications in drug delivery, biosensing, and environmental remediation. While core template type is known to influence stability, the role of polyelectrolyte layer number in governing poly(allylamine hydrochloride) (PAH) desorption remains poorly understood. This study quantitatively assessed PAH desorption from fluorescein isothiocyanate (FITC)-labeled shells of PMCs templated on CaCO_3_ or MnCO_3_ cores with 7, 9, or 13 layers under varying ionic conditions (distilled water, NaCl 0.2–3.0 M, Na_2_SO_4_ 0.005–1 M) over 168 h. Short-term incubations revealed no significant layer-dependent desorption differences for either core type. However, prolonged exposure uncovered a non-monotonic relationship for CaCO_3_-templated PMCs: 7-layer capsules exhibited high initial but limited subsequent release (<50% increase), 9-layer capsules showed minimal initial dissociation followed by maximal kinetic amplification (up to 2000% increase), and 13-layer capsules displayed intermediate behavior. In contrast, MnCO_3_-templated PMCs demonstrated uniformly low initial dissociation with gradual time- and concentration-dependent release irrespective of layer number. These findings establish core template nature as the dominant factor controlling dissociation kinetics, while layer number enables fine-tuning of release profiles—particularly for CaCO_3_ systems—providing design principles for controlled-release applications requiring delayed or sustained payload delivery.

## 1. Introduction

Polyelectrolyte microcapsules (PMCs) fabricated via the layer-by-layer (LbL) assembly method represent promising systems for addressing challenges in numerous fields of science and technology [1,2,3,4]. In pharmaceuticals, PMCs are utilized for the controlled release of bioactive compounds, where shell stability is critically important to prevent premature drug release into the bloodstream and to ensure targeted delivery to the acidic environment of tumor tissues [5]. In biosensing, PMCs serve as carriers for enzymes such as urease, glucose oxidase, alcohol dehydrogenase, and others, where their stability determines the accuracy of substrate concentration measurements, since gradual degradation of the PMC shell leads to loss of the encapsulated enzyme and a consequent reduction in sensor sensitivity [6,7,8].

In microcapsules containing photocatalysts (e.g., TiO_2_), the stability of the PMC shell prevents nanoparticle aggregation and enables repeated utilization of the system, as confirmed by studies of Shchukin et al. [9]. In environmental technologies, PMCs are applied for the sorption of heavy metal ions from aqueous solutions, where their degradation under saline conditions may result in decreased purification efficiency [10].

However, the practical application of PMCs in the aforementioned fields is limited by insufficient predictability of shell stability under operational conditions. In particular, desorption of poly(allylamine hydrochloride) (PAH) leads to uncontrolled release of encapsulated agents, which is critical for therapeutic efficacy [11]. For example, in the delivery of antitumor drugs, PMCs must maintain structural integrity in the bloodstream while degrading in the acidic microenvironment of tumor tissues [5].

It is well established that PAH desorption from the PMC shell is governed by external factors, including the ionic strength and pH of the medium. For instance, significant PAH desorption is observed in 2 M NaCl due to charge screening and consequent loosening of the shell structure [11,12]. A similar effect occurs at pH ≈ 7–8, where the amine groups of PAH undergo deprotonation with increasing pH, thereby weakening the electrostatic interactions with polystyrene sulfonate (PSS) [11,13].

Furthermore, our previous studies have demonstrated that the core template type (CaCO_3_, MnCO_3_, or polystyrene) fundamentally influences the desorption behavior of PMCs even after its complete removal [14]. PMCs templated on CaCO_3_ exhibit a sponge-like internal morphology lacking a distinct shell, whereas PMCs fabricated using polystyrene and MnCO_3_ cores form a well-defined shell structure [11,14]. These structural differences govern differential stability against ionic strength: PMCs templated on CaCO_3_ retain structural integrity at 200 mM NaCl, whereas PMCs templated on polystyrene and MnCO_3_ cores exhibit significant desorption already at 100 mM [14].

The number of polyelectrolyte layers represents a key parameter governing the thickness, density, and degree of interpenetration of polyelectrolyte chains within the PMC shell [15,16,17,18,19]. Previous studies indicate that increasing the number of layers generally enhances mechanical strength and reduces shell permeability [15,16,17,18]. Moreover, polyelectrolyte adsorption per subsequent layer typically decreases with additional layers, owing to the expanded surface interaction area. To date, however, no systematic study has examined the influence of layer number on PAH desorption across varying shell architectures and core template types. Work [19] has demonstrated that the dependence of shell thickness on the number of layers also varies with core type: for polystyrene-templated PMCs, thickness increases with layer number, whereas for MnCO_3_-templated PMCs it does not. This indicates the non-universal character of the relationship between layer number and stability across different capsule types.

In all previous studies [11,13,14], a fixed shell architecture—seven layers ((PAH/PSS)_3_PAH)—was employed, which precluded evaluation of this parameter’s contribution. Consequently, it remains unclear how the number of layers modulates PAH desorption within the context of the established dependence on core type and medium conditions.

The aim of the present study is to quantitatively assess the dependence of PAH desorption on the number of polyelectrolyte layers in the shells of PMCs synthesized on cores of different types (CaCO_3_, MnCO_3_) under various incubation conditions (ionic strength, salt type). The obtained data will enable the optimization of PMC architecture for specific operational conditions. This work concludes a research cycle dedicated to investigating the stability of polyelectrolyte microcapsules and represents a comprehensive analysis of all previously obtained results, generalizing the patterns governing PAH desorption behavior as a function of PMC structure and medium conditions.

## 2. Materials and Methods

Polystyrenesulfonate sodium (PSS) and polyallylamine hydrochloride (PAH) with a molecular mass of 70 kDa Sigma (Merck KGaA, Darmstadt, Germany), fluorescein isothiocyanate (FITC) Sigma (Merck KGaA, Darmstadt, Germany); ethylenedia-minetetraacetic acid (EDTA), dimethylformamide (DMFA), calcium chloride (CaCl_2_ × 2H_2_O), sodium chloride, sodium sulfate and sodium carbonate from Reahim (Reahim AO, St. Petersburg, Russia) were used.

### 2.1. Preparation of Fluorescently Labeled PAH

The polyelectrolyte (PAH) was labeled with FITC using a procedure adapted from our previous experiment [20], and conjugation to PAH was carried out in borate buffer (50 mM, pH 9.0). To a stirred solution (300–400 rpm) of the polyelectrolyte (10 mg/mL), FITC was introduced slowly at a molar ratio of 1:100 (FITC:PAH) [11]. This labeling reaction, conducted for 1.5–2 h at room temperature, was followed by overnight dialysis against a large volume of water (10 L) for purification of the FITC-PAH conjugate.

### 2.2. Preparation of CaCO_3_ and MnCO_3_ Microspherulites

The synthesis involved coprecipitation [11,21]. Under constant stirring, a 0.33 M solution of CaCl_2_ (or MnCl_2_) was introduced into an equal concentration (0.33 M) of Na_2_CO_3_. This rapid mixing phase lasted 30 s. Subsequently, the suspension was held static to enable complete particle precipitation and a controlled “ripening” process, tracked using light microscopy. Following established isolation procedures [20], the supernatant was decanted, and the precipitate underwent water washing prior to PMC preparation. Microparticle size analysis yielded an average diameter of 4.5 ± 1 (CaCO_3_) and 3 ± 1 μm (MnCO_3_) matching our prior synthesis.

### 2.3. Preparation of Polyelectrolyte Microcapsules Formed on CaCO_3_ and MnCO_3_

The synthesis of polyelectrolyte microcapsules (PMCs) via layer-by-layer (LbL) adsorption onto sacrificial carbonate cores followed our previously detailed procedure [22]. Microspherulites (CaCO_3_ or MnCO_3_) served as templates for the alternating deposition of PAH and PSS from aqueous solutions (2 mg/mL polymer + 0.5 M NaCl). To ensure surface cleanliness after each adsorption, templates underwent three washes with 0.5 M NaCl solution, utilizing centrifugation for separation to eliminate non-adsorbed polymer chains. After depositing the desired number of bilayers, the core was dissolved by incubating in 0.2 M EDTA for 12 h. This dissolution step initiates the formation of an interpolyelectrolyte complex within the nascent capsule interior [22]. Final purification involved triple rinsing with water to remove core remnants. PMC size distribution, analyzed by dynamic light scattering (Malvern Zetasizer Nano ZS, London, UK), yielded average diameters of 4.5 ± 1 μm (CaCO_3_-derived) and 3 ± 1 μm (MnCO_3_-derived), replicating dimensions reported in our earlier study.

### 2.4. Registration of FITC-Labeled PAH Desorption from Polyelectrolyte Capsules

Fluorescence spectroscopy analysis of microcapsule dissociation followed our established methodology [20], employing FITC-labeled PAH in one capsule layer. Samples were centrifuged (3000 rpm, 1 min), after which 10 μL supernatant was diluted 40-fold to optimize detection range. Fluorescence intensity measurements used a Cary Eclipse spectrofluorometer (Agilent) with 1 cm thermostatted cuvette (λ_ex_ = 495 nm; λ_em_ = 525 nm). Between measurements, samples were vortexed and returned for continued incubation. Spectra acquisition monitored dissociation kinetics via FITC signal intensity.

### 2.5. Statistical Data Analysis

For each measurement of fluorescence intensity, the mean values and relative standard deviations were calculated. The number of replicates (N) was 6. The significance of the differences was assessed by using a Tukey HSD post hoc test, *p* > 0.05 being considered significant.

## 3. Results

**Figure 1:**
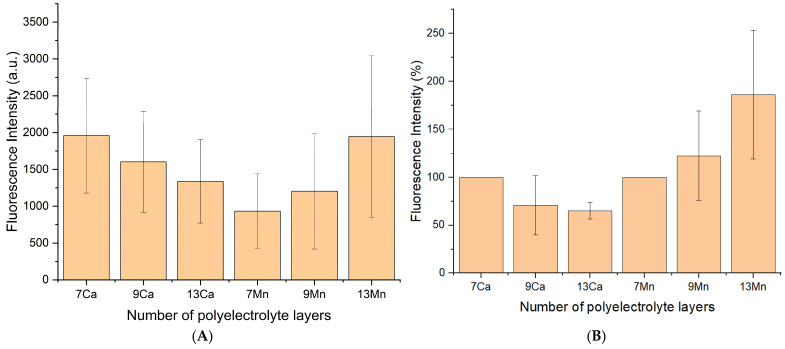
Fluorescence intensity of FITC-labeled PAH as a function of the number of polyelectrolyte layers in microcapsules templated on CaCO_3_ (Ca) and MnCO_3_ (Mn) cores. (**A**) Fluorescence intensity (arbitrary units); (**B**) Fluorescence intensity normalized to that of the 7-layer PMC sample (set to 100% of each PMC’s type (1st column)).

In the first stage of the study, we evaluated the influence of the number of polyelectrolyte shell layers on the dissociation of poly(allylamine hydrochloride) (PAH) deposited as the outermost layer during the formation of polyelectrolyte microcapsules (PMCs) by the layer-by-layer (LbL) assembly method with core removal at the final stage of PMC preparation. The experiment was performed on two capsule types: those templated on CaCO_3_ and MnCO_3_ cores, as we had previously established distinct dissociation kinetics of the shells for these systems. For this purpose, PMCs were incubated in distilled water for 7 days, followed by fluorescence measurements of the supernatant to quantify the dissociation of the fluorescently labeled PAH layer. The obtained data is presented in Figure 1.

Analysis of the results (Figure 1) revealed that, for both CaCO_3_- and MnCO_3_-templated capsules, no statistically significant differences were observed in the extent of dissociation of the outermost PAH layer when varying the number of shell layers (from (PAH/PSS)_3_PAH to (PAH/PSS)_6_PAH). According to the Tukey HSD post hoc test (*p* > 0.05 and 0.1), the number of layers in the shell does not exert a substantial influence on the stability of the fluorescently labeled PAH, either in the short or long term, for both core types. Despite the absence of significant differences among layer numbers, distinct trends in the amount of dissociated outer PAH layer were observed between PMCs templated on CaCO_3_ and MnCO_3_ cores. This indicates the dominant role of core nature in governing the dissociation kinetics of outer layers [14], whereas shell architecture within the investigated range does not represent a determining factor.

**Figure 2:**
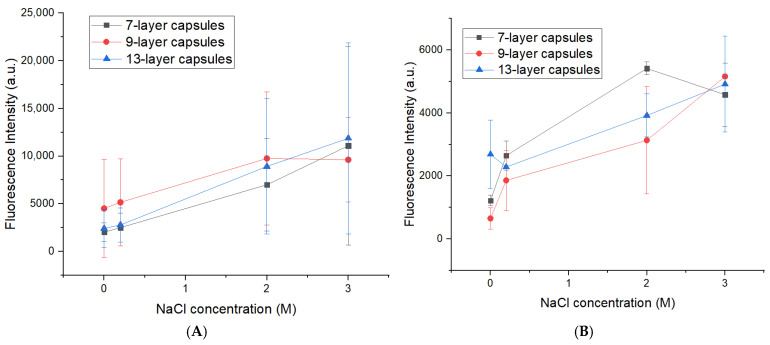
Fluorescence intensity of PAH-FITC in the supernatant as a function of NaCl concentration in solution (5 min incubation). Polyelectrolyte microcapsules templated on CaCO_3_ (**A**) and MnCO_3_ (**B**) cores.

Since the influence of shell thickness on PAH desorption proved negligible under low ionic strength conditions (distilled water), we hypothesized that altering the ionic environment might reveal underlying differences in the stability of polyelectrolyte layers. To test this hypothesis, PMCs were incubated in NaCl solutions with concentrations ranging from 0.2 to 3.0 M. It is well established that increasing ionic strength weakens electrostatic interactions between polyelectrolytes, potentially accelerating the desorption of outer layers [23]. We further hypothesized that capsules differing in shell layer number and core type would exhibit differential sensitivity to salt concentration due to variations in shell permeability, layer packing density, and the specific interactions of ions with the carbonate core [14,18]. The results are presented in Figure 2.

As evident from Figure 2A, capsules templated on CaCO_3_ cores exhibited no statistically significant differences in the extent of dissociation of the outer PAH layer, either upon variation of the number of shell layers (from (PAH/PSS)_3_PAH to (PAH/PSS)_6_PAH) or upon changing NaCl concentration (0.2–3.0 M) (Tukey HSD post hoc test, *p* > 0.05). In contrast, for PMCs based on MnCO_3_ cores, a similar independence of dissociation from shell thickness was maintained, as there was again no statistically significant difference between the groups with varying numbers of layers. However, a pronounced dependence on the ionic strength of the medium was observed: increasing NaCl concentration was accompanied by a statistically significant increase in PAH desorption (*p* < 0.05). Maximum dissociation was recorded in 3.0 M NaCl, where the fluorescence intensity of the supernatant increased by 900 ± 250% relative to the baseline level. The obtained data refute the hypothesis that the number of layers within the investigated range is not a determining factor for both capsule types.

**Figure 3:**
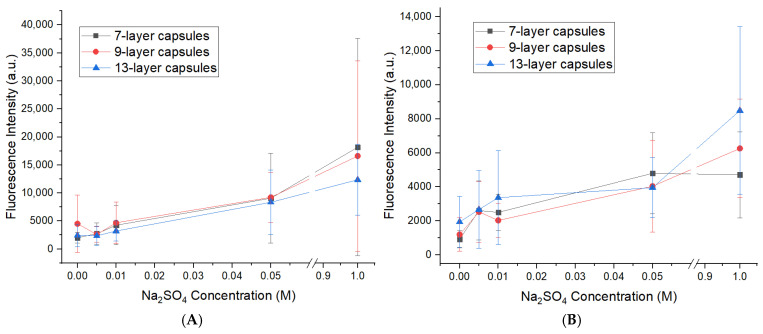
Fluorescence intensity of PAH-FITC in the supernatant as a function of Na_2_SO_4_ concentration in solution (5 min incubation). Polyelectrolyte microcapsules templated on CaCO_3_ (**A**) and MnCO_3_ (**B**) cores.

Although variation of the ionic strength using NaCl did not yield significant differences in PAH desorption among capsules with different numbers of layers (7, 9, and 13), it was hypothesized that specific ion effects might influence shell stability. In contrast to sodium chloride, sulfate ions (SO_4_^2−^) possess high charge density and are capable of cooperative binding to the amine groups of PAH, thereby enhancing desorption through competition for electrostatic interactions [13,14,20]. To evaluate this effect, PMCs were incubated in Na_2_SO_4_ solutions (0.005–1 M), followed by centrifugation and fluorescence analysis of the supernatant. The results are presented in Figure 3.

For CaCO_3_-templated capsules, no statistically significant differences in PAH dissociation were observed either among samples with different numbers of layers (7, 9, 13) or upon variation of Na_2_SO_4_ concentration (0.01–0.1 M) (Tukey HSD post hoc test, *p* > 0.05). A similar pattern was observed for MnCO_3_-templated capsules (no statistically significant differences): the number of layers did not affect dissociation (*p* > 0.05), while variation in Na_2_SO_4_ concentration led to a minor increase in supernatant fluorescence.

Thus, the number of shell layers does not determine the stability of the outer PAH layer in the presence of sulfate ions or under varying ionic strength conditions, for PMCs templated on either CaCO_3_ or MnCO_3_ cores.

To evaluate the kinetics of dissociation of the outer PAH-FITC layer, experiments were conducted with capsules templated on CaCO_3_ and MnCO_3_ cores containing 7, 9, or 13 polyelectrolyte layers under conditions identical to previous series: incubation in deionized water, NaCl solutions (0.01–2.0 M), and Na_2_SO_4_ solutions (0.005–1 M), followed by centrifugation and fluorescence analysis of the supernatant.

**Figure 4:**
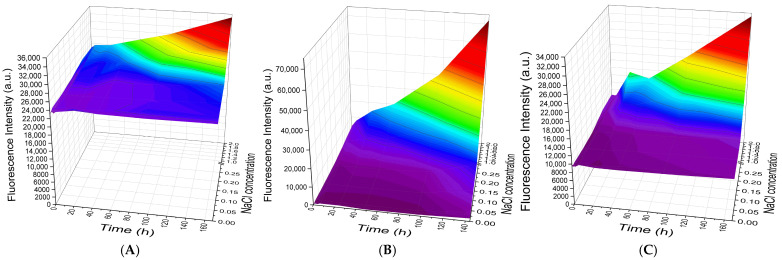
Dynamics of PAH-FITC fluorescence intensity in the supernatant following incubation of PMCs templated on CaCO_3_ cores in NaCl solutions of varying concentration. (**A**) 7-layer PMCs; (**B**) 9-layer PMCs; (**C**) 13-layer PMCs.

In the first stage, polyelectrolyte microcapsules templated on CaCO_3_ cores were investigated. The results are presented in Figure 4.

As evident from Figure 4A, dissociation of the outer PAH layer in 7-layer PMCs is already at a high level from the initial time point across all NaCl concentrations and increases by no more than 50% over 168 h of incubation. In contrast, for 9-layer PMCs (Figure 4B), dissociation commences from a value close to zero, and increasing ionic strength influences the initial amount of dissociated outer PAH layer. Over 168 h of incubation, the quantity of fluorescently labeled PAH increases by 1400%. For 13-layer PMCs (Figure 4C), an intermediate dissociation behavior is observed both at the onset of incubation and after 168 h, with a 340% increase in dissociation over the incubation period.

**Figure 5:**
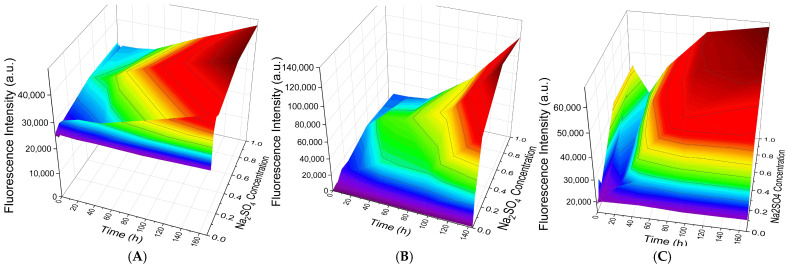
Dynamics of PAH-FITC fluorescence intensity in the supernatant following incubation of PMCs templated on CaCO_3_ cores in Na_2_SO_4_ solutions of varying concentration. (**A**) 7-layer PMCs; (**B**) 9-layer PMCs; (**C**) 13-layer PMCs.

As evident from Figure 5A, dissociation of the outer PAH layer in 7-layer PMCs is already at a high level from the initial time point across all Na_2_SO_4_ concentrations and increases by no more than 36% over 168 h of incubation. In contrast, for 9-layer PMCs (Figure 5B), dissociation commences from a value close to zero, and increasing Na_2_SO_4_ concentration affects the initial amount of dissociated outer PAH layer. Over 168 h of incubation, the quantity of fluorescently labeled PAH increases by 2000%. For 13-layer PMCs (Figure 5C), an intermediate dissociation behavior is observed both at the onset of incubation and after 168 h, with a 310% increase in dissociation over the incubation period.

**Figure 6:**
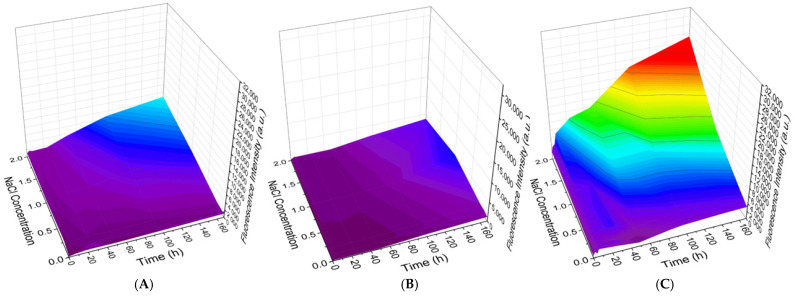

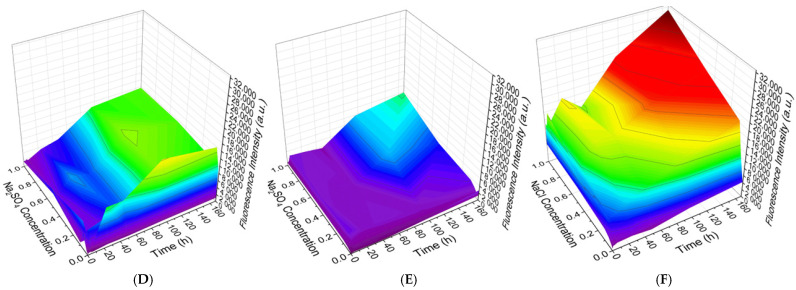
Dynamics of PAH-FITC fluorescence intensity in the supernatant following incubation of PMCs templated on MnCO_3_ cores in solutions of varying NaCl concentration (**A**–**C**) or Na_2_SO_4_ concentration (**D**–**F**). (**A**,**D**) 7-layer PMCs; (**B**,**E**) 9-layer PMCs; (**C**,**F**) 13-layer PMCs.

In the case of PMCs templated on MnCO_3_ cores, dissociation behavior distinct from that observed for CaCO_3_-templated PMCs was observed. The results are presented in Figure 6.

The dissociation kinetics of PMCs templated on MnCO_3_ matrices (Figure 6) differed substantially from those described above for CaCO_3_-templated systems. Irrespective of the number of layers (7, 9, or 13) and salt type (NaCl, Figure 6A–C; Na_2_SO_4_, Figure 6D–F), the initial degree of dissociation was negligibly small. A clear dependence was observed: both the rate and the total extent of PAH-FITC release increased with incubation time and electrolyte concentration in solution for all capsule variants examined.

Thus, the nature of the core template (CaCO_3_ vs. MnCO_3_) represents a critical factor determining the initial stability of the outer layer and the character of its dissociation kinetics in saline solutions. The results obtained for CaCO_3_-templated PMCs demonstrate a non-monotonic dependence of the outer PAH layer dissociation kinetics on the total number of layers in the PMC shell: 7-layer capsules exhibit high initial but limited overall release; 9-layer capsules show minimal initial dissociation followed by maximal kinetic increase, indicating a pronounced response to ionic strength; and 13-layer capsules occupy an intermediate position. Consequently, variation of the layer number enables deliberate tuning of the release profile—from rapid to delayed and complete—with the most controllable response observed for capsules possessing a 9-layer shell. In contrast, results for MnCO_3_-templated PMCs reveal qualitatively distinct behavior: regardless of layer number, dissociation invariably commences from near-zero values and gradually increases over time.

## 4. Discussion

The obtained results make a substantial contribution to understanding the dissociation mechanisms of polyelectrolyte microcapsules (PMCs) fabricated by the layer-by-layer (LbL) assembly method. According to literature data, an increase in the number of layers should reduce the amount of polyelectrolyte adsorbed per subsequent layer due to the enlarged surface interaction area [24], while also altering shell architecture toward denser chain packing [18,25], thereby suppressing dissociation. Moreover, increased shell thickness is generally associated with enhanced capsule stability under various conditions [18].

However, our experimental data reveal a different behavioral pattern. In short-term experiments (5 min incubation) under both low ionic strength (distilled water) and high ionic strength conditions (NaCl and Na_2_SO_4_ solutions), no statistically significant differences in the extent of dissociation of the outer PAH layer were observed upon variation of shell layer number for either core type (CaCO_3_ or MnCO_3_). This discrepancy with literature expectations can be explained by the specificities of the fabrication process for the PMCs under investigation. It is hypothesized that during the dissolution of the carbonate cores, partial intermixing of shell components occurs. This leads to structural stabilization and reduces the fraction of fluorescently labeled PAH residing in a weakly bound state susceptible to rapid dissociation. Consequently, the proportion of “weak” interactions responsible for fast desorption is substantially diminished, resulting in limited dissociation during the initial incubation period, irrespective of the total number of layers.

A more detailed investigation of dissociation kinetics over 168 h revealed an intriguing non-monotonic dependence for PMCs templated on CaCO_3_ cores. A clear correlation was observed between the number of shell layers and the PAH-FITC release profile: 7-layer capsules exhibited a high initial dissociation level that increased only marginally over 168 h (by no more than 50% for NaCl and 36% for Na_2_SO_4_); 9-layer capsules displayed minimal initial dissociation but demonstrated the maximal kinetic increase (up to 1400% for NaCl and 2000% for Na_2_SO_4_); 13-layer capsules occupied an intermediate position with moderate dissociation growth (340% for NaCl and 310% for Na_2_SO_4_).

This nonlinear effect may be explained by several factors. First, varying shell thickness may lead to differing degrees of interlayer mixing during core dissolution, which in turn affects the distribution of “weak” and “strong” interactions within the shell structure. For 7-layer capsules, formation of a less ordered structure with a greater proportion of surface-localized, weakly bound PAH molecules may occur, accounting for the high initial dissociation level. For 9-layer capsules, optimal structural compaction may take place, wherein most weakly bound PAH molecules become effectively “sealed” within the shell interior while still permitting gradual release during prolonged incubation in saline solutions. For 13-layer capsules, an excessively dense structure may form that partially restricts both the initial dissociation and subsequent PAH release. Such effects were partially demonstrated in other studies; in particular, in the work of Musin et al. [26], the effect of polyelectrolyte layer intermixing was first demonstrated, which was subsequently confirmed in the work of Dubrovskii et al. [20] for polyelectrolyte microcapsules with a large number of layers, and differences in the behavior of layer intermixing depending on the number of shell layers were demonstrated. Furthermore, in the works of Volodkin and Sukhorukov [15,17] it was demonstrated that for a number of layers less than 10, a shell is absent in PMCs formed on a CaCO_3_ core, whereas the polyelectrolyte microcapsule possesses a complex mesoporous structure resembling a sponge. Moreover, increasing the number of layers to at least 10 leads to the formation of a shell with a thickness of approximately 40 nm.

It is important to note that PMCs templated on MnCO_3_ cores exhibited a fundamentally distinct behavior: irrespective of the number of layers (7, 9, or 13) and salt type (NaCl or Na_2_SO_4_), the initial degree of dissociation was negligibly small, with a gradual increase observed upon prolongation of incubation time and elevation of electrolyte concentration. This finding corroborates the hypothesis that the nature of the core template constitutes a critical factor governing not only initial shell stability but also the dissociation kinetics of the polyelectrolyte shell. The differing dissolution rates of CaCO_3_ and MnCO_3_ in acidic media, combined with the distinct chemical properties of the released ions (Ca^2+^ versus Mn^2+^), may substantially influence the architecture and physicochemical characteristics of the polyelectrolyte shell [14]. A similar significant difference in the behavior of PMCs formed on CaCO_3_ and MnCO_3_ cores may be attributed to the fact that PMCs with an MnCO_3_ core possess a distinctly pronounced shell. Moreover, it was demonstrated in the work of Déjugnat et al. [16], that increasing the number of shell layers in PMCs does not result in shell thickening, which, according to the authors, is associated with the compaction of the PMC shell.

Thus, the obtained data demonstrate that the nature of the core template plays a more significant role in determining the dissociation kinetics of PMCs than the number of shell layers within the investigated range. Nevertheless, variation of the layer number enables deliberate tuning of the active substance release profile—from rapid to delayed and complete release—representing considerable interest for the development of controlled release systems. Particularly promising are 9-layer capsules templated on CaCO_3_ cores, which combine low initial dissociation with high capacity for subsequent active substance release, and may therefore be exploited for designing delayed-release systems under target conditions.

## 5. Conclusions

This study quantitatively assessed the dependence of poly(allylamine) desorption on the number of polyelectrolyte layers in microcapsules templated on CaCO_3_ and MnCO_3_ cores under varying ionic conditions. While short-term incubations revealed no significant layer-dependent differences for either core type, prolonged exposure uncovered a non-monotonic relationship specifically for CaCO_3_-templated systems, where 9-layer capsules exhibited minimal initial dissociation followed by maximal kinetic amplification, contrasting with the high initial release of 7-layer capsules and the intermediate behavior of 13-layer variants. In contrast, MnCO_3_-templated microcapsules demonstrated uniformly low initial dissociation with gradual time-dependent release irrespective of layer number. These findings establish the core template nature as the dominant factor governing dissociation kinetics, while confirming that layer number serves as a viable parameter for fine-tuning release profiles within CaCO_3_-based architectures.

However, it should be noted that these conclusions are based on specific layer counts and carbonate core types, and the non-monotonic behavior may depend on the specific interlayer mixing occurring during core dissolution. Future investigations should focus on elucidating the structural mechanisms driving this nonlinear effect across a broader range of polyelectrolyte pairs and core morphologies to generalize these stability patterns. Ultimately, the simultaneous optimization of core template selection and shell layer number provides a robust strategy for engineering polyelectrolyte microcapsules with predictable stability tailored to specific operational ionic environments.

## Data Availability

The original contributions presented in this study are included in the article. Further inquiries can be directed to the corresponding authors.
